# *SMARCB1* missense mutants disrupt SWI/SNF complex stability and remodeling activity

**DOI:** 10.21203/rs.3.rs-6018128/v1

**Published:** 2025-03-26

**Authors:** Garrett W Cooper, Benjamin P Lee, Won Jun Kim, Yongdong Su, Victor Z Chen, Eliseo Salas, Xiaoping Yang, Robert E Lintner, Frederica Piccioni, Andrew O Giacomelli, Thomas P Howard, Pritha Bagchi, Karen N Conneely, David E Root, Bo Liang, William C Hahn, David U Gorkin, Jaclyn A Biegel, Susan N Chi, Andrew L Hong

**Affiliations:** 1 Department of Pediatrics, Emory University School of Medicine, Atlanta, GA, USA; 2 Aflac Cancer and Blood Disorders Center - Children’s Healthcare of Atlanta, Atlanta, GA, USA; 3 Dana-Farber Cancer Institute, Boston, MA, USA; 4 Broad Institute of MIT and Harvard, Cambridge, MA, USA; 5 Department of Biochemistry, Emory University School of Medicine, Atlanta, GA, USA; 6 Humber Polytechnic, Toronto, ON, Canada; 7 Emory Integrated Proteomics Core, Emory University, Atlanta, GA, USA; 8 Department of Human Genetics, Emory University School of Medicine, Atlanta, GA, USA; 9 Department of Biology, Emory University, Atlanta, GA, USA; 10 Department of Pathology, Children’s Hospital Los Angeles and Keck School of Medicine, University of Southern California, Los Angeles, California, USA; 11 Boston Children’s Hospital, Boston, MA, USA; 12 Winship Cancer Institute, Atlanta, GA, USA

## Abstract

Chromatin remodeling complexes, such as the SWItch/Sucrose Non-Fermentable (SWI/SNF) complex, play key roles in regulating gene expression by modulating nucleosome positioning. The core subunit SMARCB1 is essential for these functions, as it anchors the complex to the nucleosome acidic patch, enabling effective chromatin remodeling. While biallelic inactivation of *SMARCB1* is a hallmark of several aggressive pediatric malignancies, the functional implication of missense mutations is not fully understood. Current diagnostic approaches focus on detecting the presence or absence of SMARCB1 by immunohistochemistry (IHC) often without consideration of mutation status as such data is lacking. Here, we present the first comprehensive deep mutational scanning (DMS) of *SMARCB1*, encompassing 8,418 amino acid substitutions, to systematically assess their functional impact. We show that missense mutations in the RPT2 domain of SMARCB1 disrupt SMARCB1 tumor suppressor function by destabilizing the SWI/SNF complex. Notably, we identify mutations in RPT2 that impair chromatin remodeling and transcriptional regulation to an extent comparable to nonsense mutations, despite maintaining detectable protein expression, thus challenging the conventional diagnostic reliance on IHC. Importantly, these mutations seem to act by disrupting winged-helix domain flexibility. These findings provide a deeper understanding of the role of SMARCB1 in chromatin remodeling and cancer biology, highlighting the limitations of current mutation classification approaches. By establishing a high-throughput functional framework, this study offers a critical resource for elucidating *SMARCB1*’s mutational landscape and its implications for cancer diagnostics.

## Introduction

The SWItch/Sucrose Non-Fermentable (SWI/SNF) chromatin remodeling complex, also known as the BRG1/BRM-associated factors (BAF) complex, regulates DNA accessibility and gene expression by positioning nucleosomes at promoters and enhancers^1^. One of the core complex subunits, SMARCB1 (also known as INI1 or BAF47), is required for regulation of enhancers^2,3^. Recent structural studies reveal that SMARCB1, in part, accomplishes this by anchoring the complex to the nucleosome acidic patch, thereby facilitating the remodeling activity of the SWI/SNF complex^4^.

Biallelic inactivation of *SMARCB1* is found in over 90% of several aggressive pediatric malignancies including malignant rhabdoid tumor of the kidney (MRTK), atypical teratoid rhabdoid tumor (ATRT), and renal medullary carcinomas (RMC)^5,6^. Rare SMARCB1-deficient cancers have also been observed in adults such as epithelioid malignant peripheral nerve sheath tumor, myoepithelial carcinoma, and poorly differentiated chordoma^7^. Diagnosis of SMARCB1-deficient cancers largely relies on the absence of detection of SMARCB1 through immunohistochemistry (IHC)^8,9^. The role of SMARCB1 in pediatric cancers has been well characterized as a tumor suppressor; however in adult cancers, its role appears more complex, with studies suggesting that SMARCB1 may also act as an oncogene in certain contexts, including liver cancer and melanoma^10,11^. Although previous studies have identified recurrent mutations like R377H that disrupt nucleosome binding^4^, the impact of these mutations on SMARCB1’s tumor suppressor function is not fully understood.

Two primary strategies have been developed to classify variants of uncertain significance (VUS). The first leverages large-scale sequencing to identify patterns of mutation recurrence^12^. For example, AACR Project GENIE v16.1 has assembled clinical sequencing data from 184,980 patients over 9 years^13^. The somatic mutation frequency of *SMARCB1* is 0.92% across 239 cancer types, excluding MRTK, ATRT, and RMC, with 1,146 missense mutations observed. In contrast, *TP53* has a much higher somatic mutation frequency of 39.6% with 57,420 missense mutations observed. This discordance underscores the limitations of recurrence-based methods for studying genes with a low somatic mutation frequency, such as *SMARCB1*. The second strategy focuses on directly assessing the functional impact of VUS through *in vitro* assays. Functional testing of low-frequency mutations is critical for clinical applications but remains underdeveloped for most cancer genes^14^.

Deep mutational scanning (DMS) provides a promising strategy to address the challenges of characterizing rare mutations. This technique systematically evaluates the functional effects of all possible variants in a gene and has been successfully applied to frequently mutated genes such as *TP53*, *BRCA1*, *KRAS*, and *EGFR*^15–18^. However, applying DMS to tumor suppressors like *SMARCB1*, which exhibit low somatic mutational frequencies, remains an underexplored avenue that could provide insights into SWI/SNF biology and inform cancer diagnostics.

In this study, we apply DMS to evaluate the functional consequences of SMARCB1 variants across its entire coding sequence. We identify missense mutations in the RPT2 domain that, despite retaining protein expression, impair SMARCB1’s tumor suppressor function by disrupting interactions within the SWI/SNF complex. Molecular dynamic modelling reveals that these mutants disrupt the flexibility of the N-terminal winged-helix domain (WHD), suggesting a novel mechanism by which SMARCB1 tumor suppressor function is disrupted. These findings imply that certain mutations result in loss-of-function (LOF) that could evade detection through current IHC based diagnostic approaches. Our findings provide essential insights into *SMARCB1*’s mutational landscape and offer a valuable resource for further research into its role in cancer.

## Results

### Mutational landscape of SMARCB1

**Figure 1:**
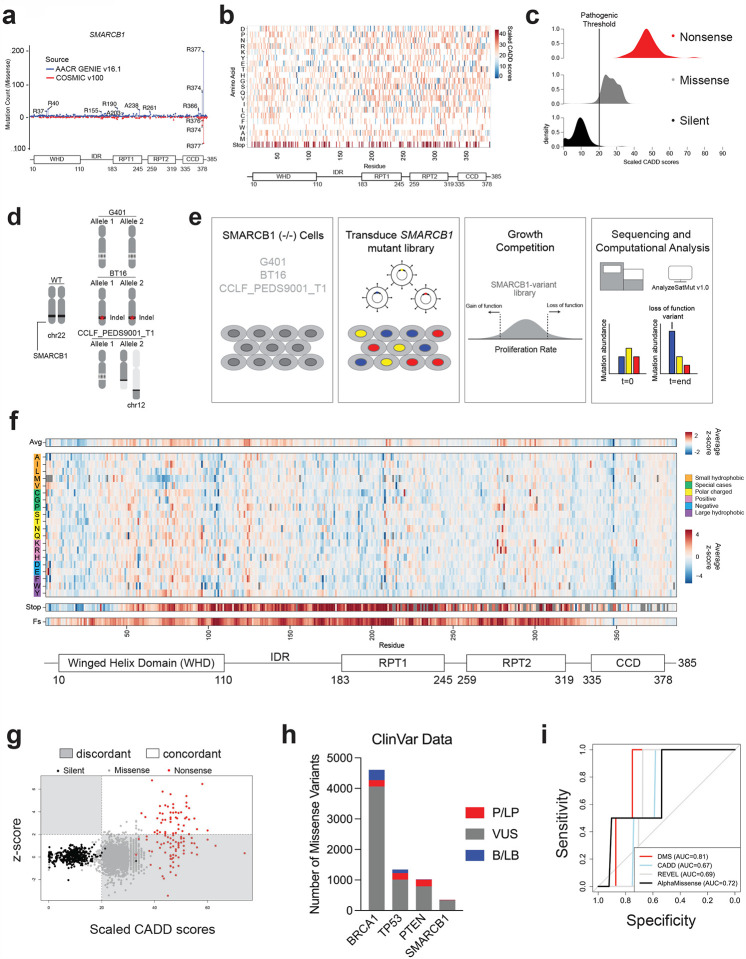
Deep Mutational Scanning of SMARCB1 Reveals Novel Regions of Mutational Intolerance in SMARCB1 **(a)** Observed mutational frequency of all cancer associated missense mutations in SMARCB1 coding sequence based on AACR GENIE v16.1 and COSMIC v100. Functional domains of SMARCB1 denoted below. **(b)** Predicted pathogenicity of all predicted variants in the SMARCB1 coding sequence from CADD v1.7. **(c)** Density plots of all mutations categorized by mutation type. Pathogenic threshold applied at a PHRED value of 20. **(d)** SMARCB1 alterations present in each cell line used in the study. G401 contains biallelic deep deletion of SMARCB1. BT16 has both a monoallelic deep deletion and a 3nt frameshift indel in exon 1. CCLF_PEDS9001_T1 has a monoallelic deep deletion and a balanced translocation in chromosome 12. **(e)** Experimental setup for deep mutational scan of *SMARCB1* including analysis steps. **(f)** Heat map representation of mutation enrichment (red) and depletion (blue) after growth competition from SMARCB1 deep mutational scanning (DMS). Values are based on three cell lines performed in biological replicates. Each column represents an amino acid position in the coding sequence of SMARCB1. The top row reflects averages of all possible 19 amino acid substitutions. In the middle heatmap, each row represents a specific amino acid substitution. The bottom two rows reflect the z-scores from stop and frameshift mutations. Missing values are denoted with a gray box. Domain structure of SMARCB1 is displayed at the bottom. **(g)** Scatterplot of all mutations SNVs predicted by CADD and their corresponding average z-score in DMS screen. Mutations colored by mutation type. **(h)** ClinVar variants (December 2, 2024 release) observed in *BRCA1, TP53, PTEN, and SMARCB1*. **(i)** AUC analysis evaluating the predictive accuracy of screen data based on cancer associated missense variants classified as pathogenic or likely pathogenic from ClinVar.

The integration and sharing of genomic and clinical data have generated a powerful resource to detect mutational trends across a broad spectrum of cancer-associated genes. We analyzed data from two leading consortia, AACR Project GENIE v16.1 and COSMIC v100, to explore recurrent missense mutations in *SMARCB1* across its key regions, including the winged helix domain (WHD), the intrinsically disordered region (IDR), the RPT1 domain, the RPT2 domain, and the coiled-coil domain (CCD) (Fig 1a, **Supplementary Table 1**). Both datasets identified low-frequency missense mutations (affecting fewer than 10 patients) distributed across the entire coding sequence of *SMARCB1*. The AACR GENIE dataset identified high-frequency mutations (affecting more than 10 patients) in 10 specific residues. In addition, both datasets identified a cluster of high-frequency mutations within the CCD, at residues R374 and R377. The most frequent of these mutations, R377H, has been shown to disrupt the remodeling function of the SWI/SNF complex; however, its role in disrupting the tumor suppressor function of SMARCB1 has not been characterized^4^.

**Extended Data Figure 1:**
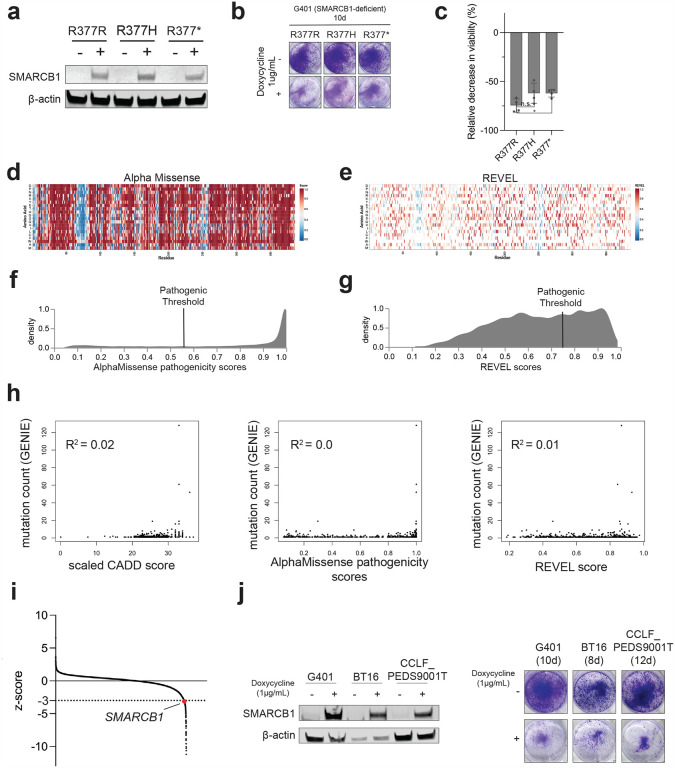
**(a)** Immunoblot showing inducible expression in the G401 cell line of the silent R377R, missense R377H, and nonsense R377*, SMARCB1 constructs after 48 hours of induction with 1 μg/mL doxycycline. **(b)** Crystal violet for each construct after 10 days of induction. **(c)** Cell counts as assessed by trypan blue exclusion after 8 days of induction (n=4). *P value < 0.05 from a Student’s two-tailed unpaired *t* test. **(d)** Predicted pathogenicity of all predicted variants in the *SMARCB1* coding sequence from AlphaMissense. **(e)** Predicted pathogenicity of all predicted variants in the SMARCB1 coding sequence from REVEL v1.3 **(f)** Density plots of all missense mutations predicted from AlphaMissense. Pathogenic threshold applied at a pathogenicity score value of 0.56. **(g)** Density plots of all missense mutations predicted from REVEL. Pathogenic threshold applied at a pathogenicity score value of 0.78. **(h)** Correlation of predicted pathogenicity and patient mutational frequency for all the computational predictors **(i)** Genes from the hORFeome V8.1 Library ORF screen in G401 cell line ranked by z-score (*SMARCB1* highlighted in red). **(j)** Immunoblot and crystal violet staining of SMARCB1 induction in G401, BT16, and CCLF_PEDS9001_T1 cell line.

To investigate the tumor suppressor function of the R377H variant *in vitro*, we used an inducible SMARCB1 re-expression system, which has previously been shown to inhibit cell proliferation when SMARCB1 is re-expressed in SMARCB1-deficient cancer cell lines^19^. We re-expressed three different SMARCB1 variants in the SMARCB1-deficient G401 cell line: the silent R377R mutant, which is expected to slow proliferation, the missense R377H mutant, and the nonsense R377* mutant (Extended Data Fig 1a, **Supplementary Table 2**). This approach allowed us to assess the functional consequences of these mutations on cell proliferation. We observed a modest, but statistically significant, decrease in cell proliferation in the R377* construct, while the R377H construct showed no significant growth difference. These results suggest that the R377* mutation partially compromises tumor suppressor function, while the R377H mutation retains its function. (Extended Data Fig 1b–c). This finding aligns with previous reports indicating that individuals with Coffin-Siris syndrome harboring germline mutations in the C-terminal CCD domain of *SMARCB1*, and specifically R377H (ClinVar Accession: VCV000030203.13), do not develop cancers at higher rates than the population^20^. Here we see that mutation recurrence does not correspond to pathogenicity within the context of this assay.

To gain further insight into the potential pathogenicity of *SMARCB1* variants, we used machine learning-based tools—CADD, AlphaMissense, and REVEL—that predict the functional impact of single nucleotide variants (SNVs) (Fig 1b, Extended Data Fig 1d–e, **respectively, Supplementary Tables 3–5**)^21–23^. We found that all three computational predictors identified a high proportion of missense variants as pathogenic based on stringent recommended thresholds: CADD (96.5%), REVEL (37.8%), and AlphaMissense (76.5%) (Fig 1c, Extended Data Fig 1f–g). This finding contrasts with the low somatic missense mutation frequency of *SMARCB1* in patient tumor samples (0.60%) based on the GENIE dataset. If such a large proportion of variants were truly pathogenic, we would anticipate a higher frequency of missense mutations in *SMARCB1* in patients. Furthermore, while we see that CADD correctly predicts the pathogenicity of recurrent mutations such as R377H, R377C, and R374Q, we do not see that predicted pathogenicity correlate with observed patient frequency (Extended Data Fig 1h). Given the discordance of our *in vitro* studies on the recurrent R377H mutation and the fact that these computational tools predict pathogenic effects broadly outside a cancer-specific context, we sought to investigate the functional effects of all possible missense mutations in *SMARCB1* using DMS.

### Deep mutational scanning of SMARCB1 coding sequence

To validate the feasibility of a large-scale open reading frame (ORF) screen in *SMARCB1*-deficient cell lines such as a DMS screen, we introduced the hORFeome V8.1 Library composed of 16,100 ORF’s, including *SMARCB1*, in the G401 cell line (Methods)^24^. Our results showed that cells with wild type *SMARCB1* were negatively selected with a z-score of −3.12 (Extended Data Fig. 1i, **Supplementary Table 6**). We then generated a deep mutational scanning (DMS) library of *SMARCB1* variant 1 (ENST00000644036.2), the predominant wild-type (WT) isoform, comprising 8,418 variants (corresponding to 24,709 nucleotide variants) including silent, missense, frameshift, and nonsense mutations (Methods, **Supplementary Table 7**). We employed a proliferation-based DMS approach to model *SMARCB1* variant function in 3 patient-derived SMARCB1-deficient cell lines: G401 – MRTK (biallelic deletions encompassing *SMARCB1*), BT16 – ATRT (biallelic indel in exon 1), and CCLF_PEDS9001_T1 – RMC^19^ (monoallelic deletion with a disruptive balanced translocation in *SMARCB1*) (Fig 1d, Extended Data Fig 1j).

### Evaluation of SMARCB1 function using DMS

We introduced the *SMARCB1* mutant library into the three SMARCB1-deficient cell lines at a low multiplicity of infection (0.2–0.5) (Fig 1e). Fitness of these mutants based on proliferation was carried out for 8–14 days, and Analyze Saturation Mutagenesis v1.0 was used to assess variant abundance following fitness competition^25^. A functional z-score for each variant was calculated by comparing the log2 fold change of the variant to that of its corresponding silent mutant (Fig 1f, Methods). Variants with a z-score >2 were considered LOF. Interactive versions of these heatmaps and data processing pipelines are available at https://github.com/ahonglab/SMARCB1_DMS.

**Extended Data Figure 2:**
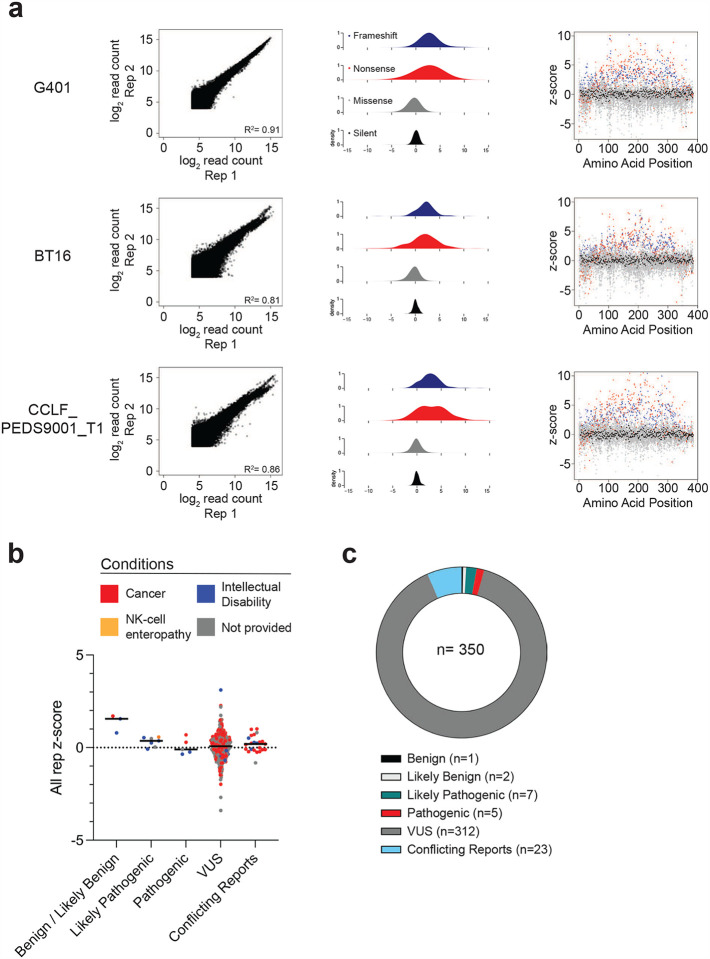
**(a)** Correlation of all mutant counts in the DMS library for each cell line. Density plots and mutation specific z-scores for each mutation type are shown. **(b)** ClinVar predicted missense variants colored by associated disease. **(c)** Circle plot depicting proportion of each category of ‘Germline’ classification as assessed through ClinVar.

We then combined data from these three cell lines to identify alleles that scored in more than one cell line (Extended Data Fig 2a). Specifically, z-scores for each variant were averaged across the three cell lines to account for variability between these models with differing modes of SMARCB1 disruption (Figure 1d). Variants with a low standard deviation across the three contexts were prioritized for further analysis as these represent consistent functional impacts regardless of the cellular background. We found that integrating our DMS dataset with the corresponding SNVs from the CADD dataset revealed largely concordant predicted functional outcomes for silent and nonsense mutants (Fig 1g, **Supplementary Table 8**). Specifically, 44.1% (52/118) of nonsense mutants showed concordant functional predictions. Similarly, both datasets concordantly identified 99.18% (362/365) of silent mutants as non-pathogenic. However, for missense mutations, there was significant discordance, with only 4.18% (95/2274) of these mutations having concordant functional predictions between the datasets.

To contextualize our data within the framework of currently recognized pathogenic mutations, we examined the number of established pathogenic missense mutations in three well-characterized tumor suppressors, *BRCA1*, TP53, and *PTEN*, and compared to those in *SMARCB1* (Fig 1h, Extended Data Fig 2b–c). We found that only 12 missense mutations in *SMARCB1* were classified as pathogenic or likely pathogenic, with two of these directly linked to cancer. The remaining mutations were associated with intellectual disability (n=6), NK-cell enteropathy (n=1), or not provided (n=3). In comparison, *BRCA1*, *TP53* and *PTEN* had a higher number of missense variants classified as pathogenic/likely pathogenic than *SMARCB1*. Specifically, *BRCA1* had 3.6% (209/5807) pathogenic variants, 203 of which were linked to cancer pathogenesis; *TP53* had 15.9% (214/1348) with 212 variants associated with cancer; and *PTEN* had 21.75% (223/1025), with 148 associated with cancer **(Supplementary Table 9)**. Since our screen was conducted in cancer cell lines to assess disruption of tumor suppressor function, we focused our receiver operating characteristic (ROC) analysis on pathogenic missense mutations known to be linked with cancer as true positives (Fig 1i). In comparing the performance of various computational predictors to our functional assay, we observed that our DMS data (AUC=0.81) outperformed all other predictors: CADD (AUC=0.67), REVEL (AUC=0.69), and AlphaMissense (AUC=0.72).

### Functional scores of 8,418 SMARCB1 mutants

**Figure 2:**
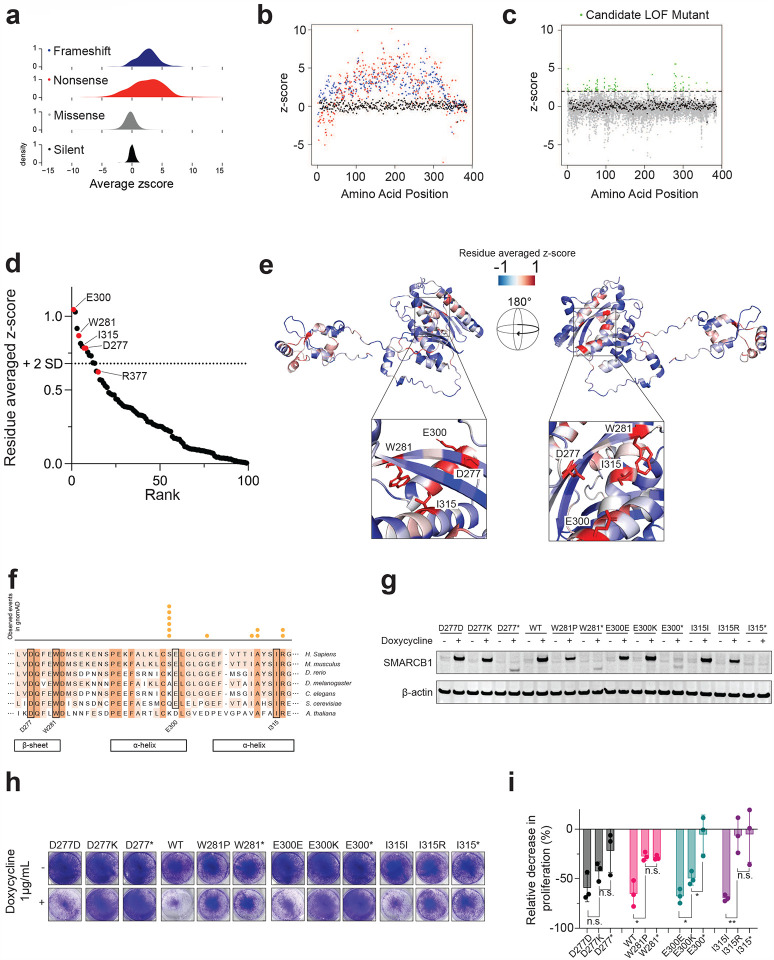
*In vitro* Validation of Top DMS Hits Reveals Novel Missense Mutants Drive LOF Phenotype **(a)** Density plots showing the functional z-score distribution averaged across all replicates of screen across the 4 different types of mutations: frameshift, nonsense, missense, and silent. A functional z-score > 0 denotes mutation enrichment and < 0 denotes mutation depletion compared to silent mutations **(b)** Averaged functional z-score across all replicates for silent (in black), frameshift (in blue), and nonsense mutations (in red) across the length of the *SMARCB1* coding sequence. **(c)** Averaged functional z-score across all replicates for silent (in black) and missense mutations (in grey) across the length of the *SMARCB1* coding sequence. Candidate LOF missense mutations with an average functional z-score above 2 are highlighted in green. **(d)** Residue rank plot showing the residue average functional z-score of all possible 19 amino acid substitutions for all residues with a positive value (n=99). Top hits in the RPT2 domain and recurrent cancer associated R377 residue are labeled in red. **(e)** Structural mapping of residue average functional z-scores focusing on top intolerant residues in RPT2 domain on computationally predicted structure of SMARCB1 from AlphaFold v2.0. Image has been rotated 180° to further visualize interactions. Zoomed interaction of intolerant RPT2 intolerant residues is depicted. **(f)** Evolutionary conservation of residues 277 to 317 of SMARCB1 across 7 eukaryotic species. Number of observed missense mutations in gnomAD v2.1.1 depicted above. **(g)** Immunoblot showing inducible expression from total protein lysates in the G401 cell line of the wildtype, missense mutant or nonsense mutant for residues D277, W281, E300, and I315 after 48 hours of induction with 1ug/mL doxycycline. **(h)** Crystal violet staining of G401 cells after 10 days of induction. Re-expression of WT SMARCB1 is also included in Extended Data 1j. **(i)** Cell counts as assessed by trypan blue exclusion after 8 days of induction in G401 cell line for each construct (n=3). *P value < 0.05, **P value < 0.005 from a Student’s two-tailed unpaired *t* test.

To understand how each mutation type (silent, missense, frameshift, and nonsense) impacted SMARCB1 function in our screen, we plotted the distribution of functional scores across mutation types (Fig 2a). As anticipated, nonsense and frameshift mutants generally exhibited increased fitness compared to the baseline WT function, consistent with the expectation that truncated proteins often lack functional activity^26^. We observed that both N-terminal and C-terminal nonsense mutants showed either full or partial functional activity (Fig 2b). The residual activity observed in the N-terminal nonsense mutants is likely due to downstream methionine start sites at residues 1–4, 27, and 38 in the N-terminus of SMARCB1, which enable downstream read-through. This finding aligns with reduced efficiency of nonsense-mediated decay (NMD) observed within 200 nucleotides of start codons^27^. Additionally, the absence of functional frameshift mutants in this N-terminal region further supports this explanation. C-terminal nonsense and frameshift mutants starting at residue 350 appear to retain partial functionality likely because truncations do not remove critical functional domains necessary for its tumor suppressor activity. This observation is consistent with the previously demonstrated partially functional R377* mutant which had a z-score of 0.296. Unlike CADD predictions, we found that missense mutations largely overlapped with silent mutations but exhibited a wider distribution (Fig 2a). This observation suggests that while most missense mutations do not impact SMARCB1 function, a small subset may have a disruptive effect.

To prioritize specific candidate LOF missense mutations, we applied the LOF z-score threshold of >2.0, identifying 101 variants (Fig 2c, **Supplementary Table 10**). Notably the recurrent R377H missense mutant did not meet this threshold with a z-score of 0.985. To more broadly identify residues which were intolerant to missense mutations, each residue was ranked by averaging z-scores across the 19 possible missense mutations. Using a cutoff of two standard deviations above the mean, residues with an average functional z-score exceeding 0.679 were classified as mutation-intolerant. Among the 13 residues meeting this criterion, six were located within the WHD domain (positions: R52, A55, I63, K77, L90, and L91), three within the IDR (positions: E122, Q123, and A125), and four within the RPT2 domain (positions: D277, W281, E300, and I315) (Fig 2d, **Supplementary Table 11**).

### Functional and structural insights in mutation-intolerant regions of SMARCB1

**Extended Data Figure 3:**
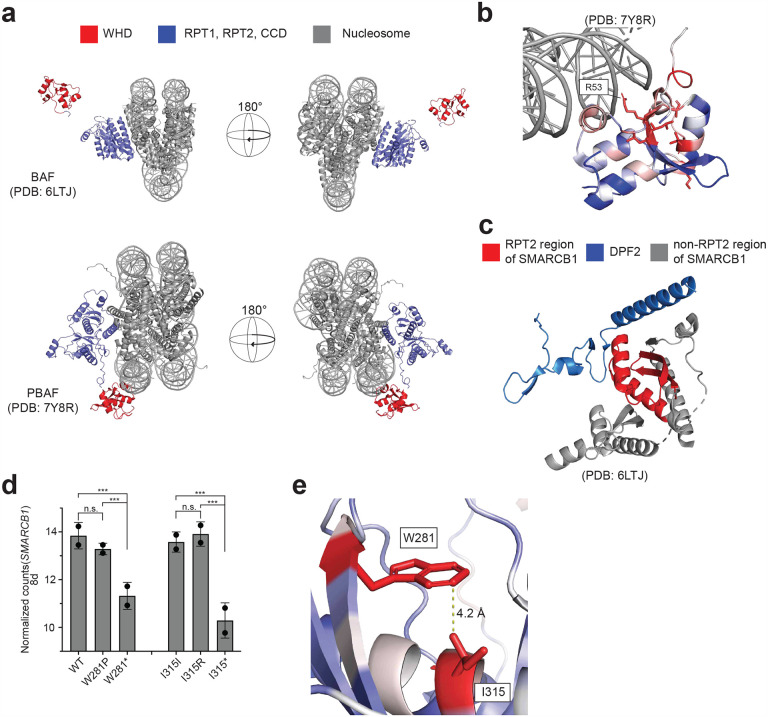
**(a)** Structural analysis of the two SMARCB1 containing SWI/SNF subfamilies, cBAF and PBAF, showing SMARCB1 is proximal to the nucleus. **(b)** PBAF cryo-EM structure (PDB: 7Y8R) of WHD binding to DNA with residue averaged z-score overlaid. **(c)** Interaction of RPT2 domain of SMARCB1 with DPF2 as described in the cryo-EM structure of cBAF (PDB: 6LTJ). **(d)** *SMARCB1* transcript abundance for each condition as assessed through bulk RNA-seq after 8 days of induction with 1μg/mL doxycycline induction. ***P value < 0.0005 from differential expression analysis using DESeq2. **(e)** Close interaction of residues W281 and I315R with residue averaged z-score overlaid on AlphaFold predicted structure of SMARCB1.

The first of these regions, the WHD, adopts distinct conformations in the two SMARCB1-containing subfamilies of SWI/SNF complexes, cBAF and PBAF, with the WHD being distal to the nucleosome in cBAF^28^ and proximal to the nucleosome in PBAF^29^ (Extended Data Fig 3a). Mutation intolerant residues, analyzed in the context of the cryo-EM-resolved PBAF structure, were found to closely interact with DNA, particularly R53 which is predicted to insert into the major groove of DNA through a charge-charge interaction, suggesting that mutations at these sites may disrupt SMARCB1-DNA interactions (Extended Data Fig 3b). Given the incomplete resolution of certain residues in available cryo-EM SMARCB1 structures particularly in the IDR, we used the AlphaFold v2.0 computationally-predicted SMARCB1 structure to comprehensively map mean functional scores for all possible missense mutations^30^. SMARCB1 consists of two globular functional domains—one at each terminus—connected by an IDR. The cluster of intolerant residues in the IDR (aa122, aa123 and aa125) did not display obvious functional characteristics; however IDRs have been known to facilitate conformation flexibility to facilitate transient protein-protein interactions and complex assembly^31^. Mutations in this intolerant cluster may impair the distal and proximal nucleosome interactions of the WHD. The last cluster of intolerant residues appear within the RPT2 domain. RPT2 is required for DPF2 association^32^ likely due to its close interaction with SMARCB1 (Extended Data Fig 3c). We observed that these intolerant residues in RPT2 interact in 3-dimensional space suggesting they may facilitate an important intramolecular interaction (Fig 2e). This region exhibits high evolutionary constraint based on its strong conservation across eukaryotic species and intolerance to missense mutations in healthy individuals, as reported in gnomAD v2.1.1^33^ (Fig 2f). Based on these observations, we then assessed the mechanism of these candidate LOF missense mutants in the RPT2 domain.

To evaluate the functional impact of the top-hit missense variants within the RTP2 domain (D277K, W281P, E300K, and I315R), we transduced G401 cell lines with an inducible vector with the corresponding silent/wildtype (hereafter referred to as wildtype), missense, or nonsense variants for each residue. In this context, ‘wildtype’ specifically refers to the re-expressed SMARCB1 ORF rather than the parental cell line. While the DMS screen used cell lines from three SMARCB1-deficient cancer contexts to ensure generalizability, subsequent functional assays were focused on the G401 model to study the effects of SMARCB1 re-expression. Using total protein lysates, we detected protein expression in both the wildtype and missense mutants as compared to the uninduced samples (Fig 2g). We then assessed the proliferation of each construct by re-expressing each construct over a 10-day period, mimicking the duration of the DMS screens. Crystal violet staining showed a distinct growth advantage in cells re-expressing missense mutant SMARCB1 constructs compared to the wildtype (Fig 2h). In a similar proliferation assay over 8 days, re-expression of the W281P, E300K, and I315R mutations conferred a significant proliferation advantage relative to wildtype controls, while D277K exhibited a modest yet non-significant proliferation advantage, corroborating the DMS results (Fig. 2i, **Supplementary Table 12**). The most pronounced effects were observed in the W281P and I315R mutants, with no significant proliferation differences between these missense mutations and their corresponding nonsense mutations. We performed bulk RNA sequencing (RNA-seq) and analyzed transcript abundance for residues W281 and I315 in each condition after 8 days of induction. Both W281P and I315R mutants exhibited *SMARCB1* transcript levels comparable to the wild type, confirming that the observed transcriptional impairments are not driven by differences in SMARCB1 expression (Extended Data Fig 3d). Structural modeling reveals that residues W281 and I315 are positioned within 4.2 Å of each other (Extended Data Fig 3e). These data collectively suggest that despite robust RNA and protein expression, the W281P and I315R mutants functionally mimic nonsense mutations.

### Mutations in SMARCB1 disrupt structural flexibility and SWI/SNF complex integrity

**Extended Data Figure 4:**
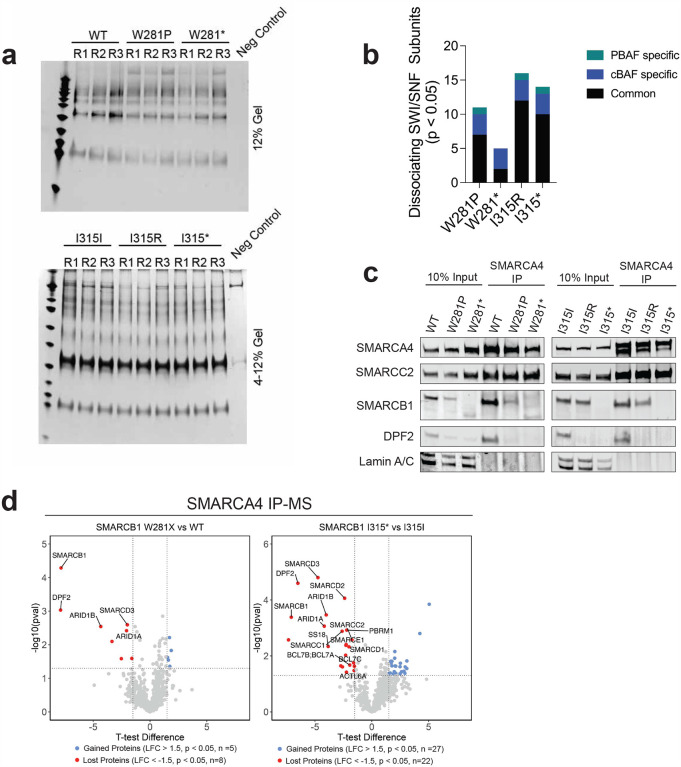
**(a)** Silver staining of mass spec inputs. **(b)** Number of dissociated subunits in each comparison of mass spectrometry data. Dissociated subunits specific to either cBAf or PBAF are indicated in the legend. **(c)** Immunoblots of IP showing complete dissociation of DPF2 in the W281P and I315R mutant conditions. **(d)** Volcano plots of mass spectrometry data showing significant proteins (in red or blue) observed when comparing the nonsense mutants to the wildtype constructs.

**Figure 3:**
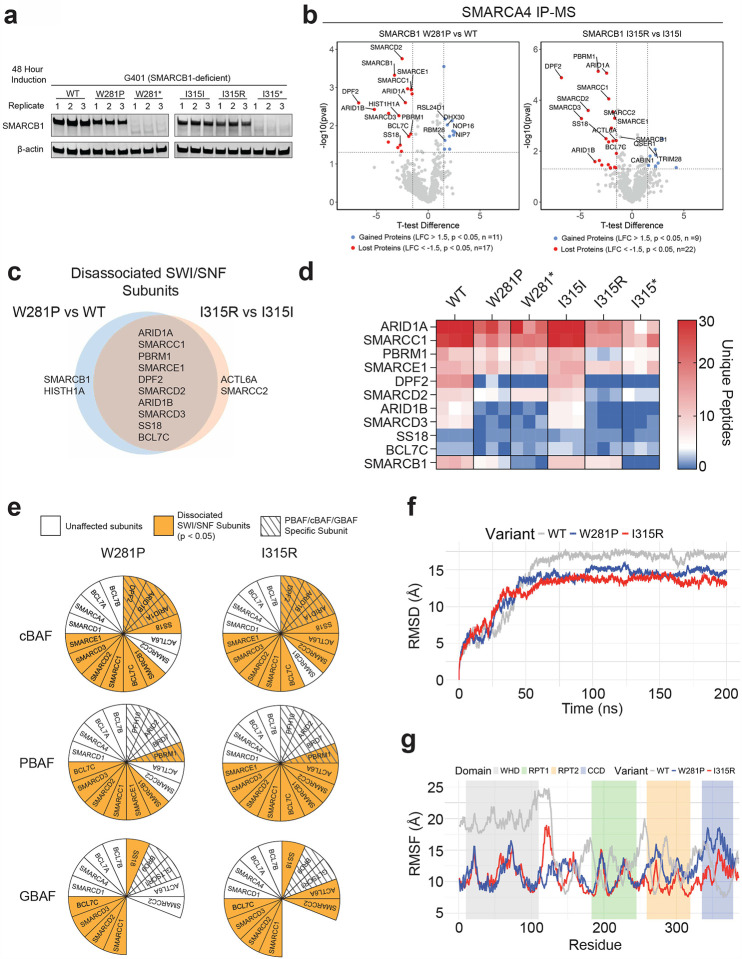
Top DMS Hits Lead to SWI/SNF Complex Destabilization **(a)** Immunoblot from nuclear extracts from three biological replicates that were loaded as input into the SMARCA4-IP and submitted for mass spectrometry analysis. **(b)** Volcano plot of proteins significantly depleted (red) and enriched (blue) in the mutant condition compared to the wildtype. SWI/SNF subunits are labeled. **(c)** Venn diagram showing overlap of SWI/SNF subunits which were depleted in both mutant conditions. **(d)** Unique peptide counts for all overlapping SWI/SNF subunits across three biological replicates for all 6 conditions. **(e)** Graphic representing which subunits from each SWI/SNF subfamily showed significant depletion in missense mutant conditions. **(f)** RMSD plot from 200ns molecular dynamics simulation for the wildtype, W281P, and I315R mutated structures. **(g)** RMSF plot of positional fluctuation for each residue at the end of the 200ns simulation for each construct.

To evaluate the effects of the W281P and I315R mutations of SMARCB1 on SWI/SNF complex stability, we performed SMARCA4 co-immunoprecipitation (co-IP) followed by mass spectrometry using nuclear protein extracts after 48 hours of induction (Fig 3a; Methods; Extended Data Fig 4a). SMARCA4 (BRG1) is a core, stable subunit of the SWI/SNF complex involved in chromatin remodeling^34^. Targeting SMARCA4 allows for reliable detection of complex stability, even when the SMARCB1 antibody may not efficiently capture the complex due to mutations or subunit instability. Among the proteins significantly enriched in the mutant conditions, there were no overlaps, suggesting mutant-specific functions (Figure 3b; **Supplementary Tables 13–14**). The W281P mutant showed enhanced ribosome biogenesis (RBM28, RSL24D1, DHX30, NOP16, and NIP7), indicative of a shift toward increased cellular growth and protein production. In contrast, the I315R mutant showed an enrichment of epigenetic regulators (CABIN1, TRIM28, and QSER1), indicating a shift towards a modified chromatin state. These results highlight potential mutation-specific mechanisms contributing to tumorigenesis through altered transcriptional programs or epigenetic reprogramming.

Among the proteins significantly reduced in the mutants, both showed a loss of SWI/SNF subunits, with 11 subunits absent in the W281P mutant and 12 in the I315R mutant. (Fig 3b, Extended Data Fig 4b, **Supplementary Tables 13–14**). Of the ten subunits depleted in both missense mutants, four (ARID1A, ARID1B, PBRM1, and SMARCC1) have been found to be recurrently mutated in various cancers (Fig 3c–d, **Supplementary Table 15**)^1^. In concert with prior findings, we observed a complete or near-complete loss of DPF2 association with the complex^32^ (Fig 3d, Extended Data Fig 4c). Similar dissociation of SWI/SNF subunits - particularly ARID1A, ARID1B, and DPF2 - was also observed when comparing respective nonsense mutants to wildtype (Extended Data Fig 4d, **Supplementary Tables 16–17**).

This complex instability primarily affects the two SMARCB1-containing subfamilies of SWI/SNF complexes (cBAF and PBAF). cBAF preferentially binds to distal enhancers and PBAF preferentially binds to promoters^35^. In both mutants, we observed significant dissociation of the cBAF specific subunits, DPF2 and ARID1A/B, and the PBAF specific subunit, PBRM1 (Fig 3e). However, the effects on PBAF stability in both mutants appear to be less pronounced, as the PBAF specific subunits BRD7, ARID2, and PHF10 remain stably bound to the complex. As expected, the SMARCB1-absent SWI/SNF complex (GBAF) has no dissociation of its specific subunits BRD9 and GLTSCR1 (Fig 3e). These findings underscore that the W281P and I315R mutations in SMARCB1 disrupt the assembly and stability of the SMARCB1-containing SWI/SNF complex subfamilies which likely contributes to their nonfunctional phenotype and highlights their potential role in tumorigenesis.

We next performed molecular dynamics simulations to examine the impact of these mutations on the conformation of SMARCB1. Our analysis revealed that the overall structural deviation, as determined by root mean square deviation (RMSD), was lower in the I315R and W281P mutants relative to the wild-type conformation (Fig 3f, **Supplementary Table 18**). Further, an assessment of positional fluctuations for each residue, calculated using root mean square fluctuation (RMSF), revealed that residues 1–125 exhibited substantial fluctuation in the wild-type protein, suggesting rigid-body motion within the N-terminal WHD of SMARCB1 (Fig 3g, **Supplementary Table 19**). However, this rigid body motion of residues 1–125 is lost in both the I315R and W281P mutants. Visual analysis of this trajectory reveals that WT SMARCB1 exhibits a significant upward shift of the N-terminal WHD, reflecting its dynamic conformational flexibility. In contrast, the I315R and W281P mutants maintain a more rigid structure, suggesting impaired dynamics **(Supplementary Video 1)**. These data suggest that both mutations compromise the structural flexibility of the WHD, thereby disrupting critical protein-protein interactions within the SWI/SNF chromatin remodeling complex. Of note, the cluster of intolerant IDR residues 122–125 occur in this putative hinge region, suggesting that these IDR mutations may impair structural flexibility in a manner similar to the I315R and W281P mutants.

### Mutations impair SWI/SNF mediated chromatin remodeling and transcriptional regulation

To further explore the downstream consequences of the observed SWI/SNF complex instability in these mutants, we performed ATAC-seq to evaluate global changes in chromatin remodeling. Prior studies have demonstrated that re-expressing SMARCB1 in SMARCB1-deficient cell lines leads to global increases in SWI/SNF occupancy and chromatin accessibility particularly at distal regulatory regions^2,3^. In the comparison of WT/W281*, we observed 34,918 gained peaks and 876 lost peaks with statistical significance (FDR < 0.05). Here ‘gained’ peaks refer to regions showing increased chromatin accessibility in the wild-type context, where SMARCB1 is re-expressed, as compared to the mutant context. Similarly, in the I315I/I315* comparison, 29,459 gained peaks and 1,256 lost peaks were observed (FDR < 0.05). To comprehensively capture all affected regions, we combined the gained and lost peak sets from both comparisons (Methods). This analysis yielded a unified dataset of 40,902 gained peaks and 1,997 lost peaks, representing broad accessibility changes associated with nonsense SMARCB1 constructs.

**Figure 4:**
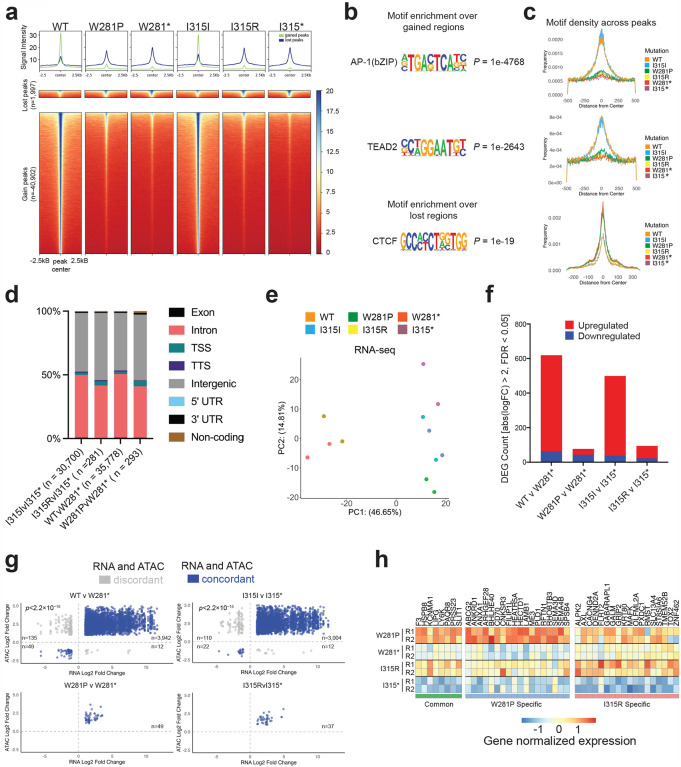
Missense mutations disrupt chromatin remodeling and transcriptional regulation **(a)** ATACseq signal across all mutant conditions at all significantly differentially accessible peaks when comparing wildtype to nonsense mutants. **(b)** Motif enrichment analysis for all gained and lost peaks depicted in volcano plots. **(c)** Motif density plotted for each motif across all six conditions. **(d)** Annotated genomic regions for all significant peaks across each comparison. **(e)** Principal component analysis of bulk RNA sequencing for all constructs. **(f)** Number of differentially expressed genes across each comparison based on RNA sequencing. **(g)** Scatterplot showing the log2 fold change (L2FC) for each significantly differentially accessible peak and its associated annotated gene for each comparison. The data includes only peaks and genes with an adjusted p-value (padj) < 0.05 and an absolute L2FC > 1. **(h)** Normalized counts from RNA sequencing for genes which showed concordant upregulation in both ATAC and RNA datasets in the mutant W281P and I315R conditions. Each row was normalized independently.

We then analyzed ATAC-seq signal intensity for wildtype, missense, and nonsense mutants across these genomic regions. SWI/SNF remodeling activity was significantly reduced at gained regions in the presence of W281P and I315R mutations, highlighting its impact on chromatin accessibility and remodeling function of the complex (Fig 4a). Sites with gained chromatin accessibility were enriched for transcription motifs such as AP-1 and TEAD2, while sites with lost accessibility were enriched for the insulator protein CTCF, consistent with previous reports (Fig 4b–c)^2,29,37^. We observed that the vast majority (I315I/I315*= 94.99%, I315R/I315*= 93.95%, WT/W281*= 94.85%, and W281P/W281*= 91.81%) of differential accessible regions in all conditions were found in intergenic and intronic regions aligning with the observations that the stability of cBAF, rather than PBAF, is predominantly disrupted (Fig 4d, **Supplementary Table 20**).

**Extended Data Figure 5:**
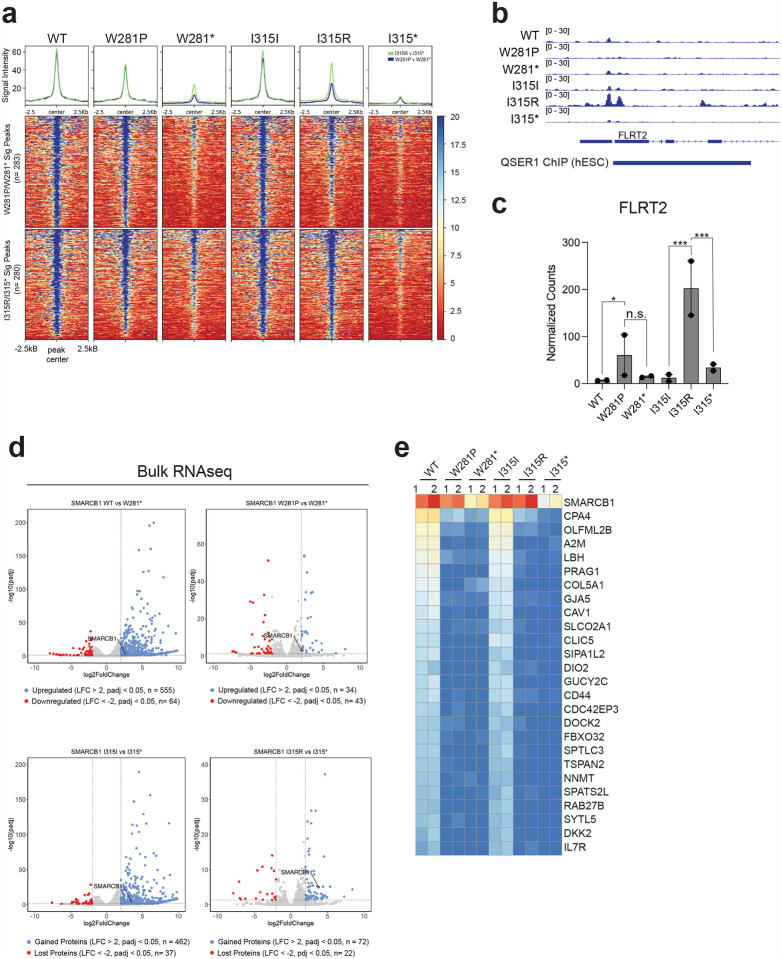
**(a)** ATACseq signal in peaks gained upon W281P or I315R expression when comparing to corresponding nonsense mutants. **(b)** ATACseq signal tracks for I315R specific gained peaks on the promoter of *FLRT2*. **(c)** Transcript abundance of FLRT2 across all six conditions as assessed by bulk RNAseq. * P value < 0.05, ***P value < 0.0005 **(d)** Volcano plots of DEGs found upon comparison of both wildtype and missense to the nonsense construct. Data summarized in Fig 4f. **(e)** Transcript abundance across all six conditions when looking at the top 25 most highly differentially expressed genes when comparing I315I to I315*.

Motif density calculations revealed minimal enrichment of AP-1 motifs in both the W281P and I315R mutants, suggesting that partial SWI/SNF remodeling function persists at a small subset of AP-1 associated sites (Fig 4c). We found 283 and 280 gained peaks in the W281P/W281* and I315R/I315* comparisons respectively. Most of these peaks exhibit similar changes in accessibility in WT context (Extended Data Fig 5a) indicating that neither mutant confers substantial novel SWI/SNF targeting. We did however identify a notable instance of I315R-specific remodeling activity at the gene *FLRT2* (Extended Data Fig 5b), a reported inhibitor of cell senescence^38^, which was accompanied by an I315R-specific increase in FLRT2 expression (Extended Data Fig 5c). Proteomic analysis showed increased SWI/SNF association of QSER1, a known inhibitor of DNA methylation at bivalent promoters^39^, exclusively in the I315R mutant. This enhanced accessibility overlapped with known QSER1 binding sites in human embryonic stem cells (hESCs), suggesting a potential unique function for the I315R mutant, possibly through QSER1-mediated remodeling of *FLRT2*.

To evaluate whether these defects in chromatin accessibility impact transcriptional regulation, we performed bulk RNAseq for each condition. Principal component analysis (PCA) revealed clear transcriptional distinctions between wildtype and mutant samples, with the I315R and W281P mutants clustering closely with their corresponding nonsense mutants (Fig 4e). Re-expression of wildtype SMARCB1 resulted in 619 significantly differentially expressed genes (DEGs) compared to the W281* nonsense mutant, whereas the W281P missense mutant yielded only 77 significant DEGs (Fig 4f, Extended Data Fig 5d). Similarly, re-expression of the silent I315I mutant led to 499 DEGs compared to the I315* nonsense mutant, while the I315R missense mutant exhibited 94 significant DEGs (**Supplementary Table 21**). The upregulation of genes typically observed with wild-type SMARCB1 re-expression was consistently disrupted across mutant conditions (Extended Data Fig 5e).

We then evaluated whether changes in chromatin accessibility resulted in corresponding transcriptional changes in target genes. We found that most significant peaks exhibited concordant changes. Specifically, the WT/W281* and I315I/I315* comparisons showed 96.5% (3991/4138) and 96.1% (3026/3148) concordance, respectively (Fig 4g, **Supplementary Tables 22–25**). Further analysis revealed that the missense mutants induced distinct patterns of transcriptional upregulation: W281P and I315R resulted in 49 and 37 concordant changes, respectively. 18 genes were unique to each mutation, while 8 genes were commonly upregulated in both mutants (Fig 4h).

**Figure 5:**
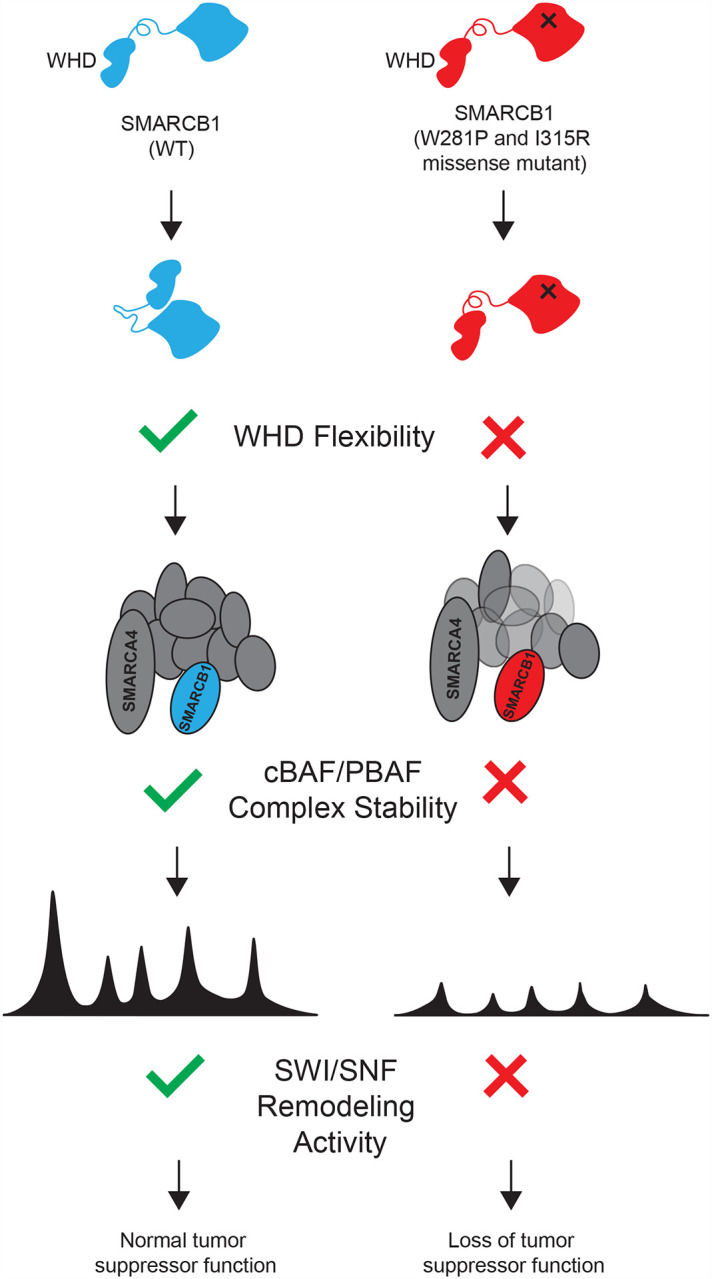
Model Proposed model for how missense mutant in RPT2 disrupts SMARCB1 function.

In summary, these data suggest that the W281P and I315R missense mutations in SMARCB1 impair SWI/SNF complex remodeling activity, affecting chromatin accessibility and transcriptional regulation at target genes (Fig 5).

## Discussion

This study employs a deep mutational scanning (DMS) approach to explore the functional landscape of *SMARCB1*, a tumor suppressor, in the context of SMARCB1-deficient cancer cell lines. Our results provide new insights into the mutational tolerance and structural vulnerability of SMARCB1, identifying critical functional domains and key residues that govern the tumor suppressor activity of SMARCB1.

Our findings highlight how the winged helix domain (WHD), intrinsically disordered region (IDR), and RPT2 domain mediate SMARCB1’s functional activity. Paired with the WHD’s DNA-binding activity, the IDR’s flexibility, and the RPT2 domain’s role in complex assembly, these findings underscore the multifaceted nature of SMARCB1’s function within the SWI/SNF chromatin remodeling complex. We saw retention of partial activity in both N-terminal and C-terminal truncations which mirrors recent DMS studies in *TP53*^15^. This retention may reflect the functional flexibility of SMARCB1, particularly its ability to utilize multiple methionine start sites in the N-terminus or retain some function in the absence of key domains.

Of particular interest are the missense mutations in the RPT2 domain, specifically W281P and I315R. These mutants exhibited functional properties akin to nonsense mutations despite robust RNA and protein expression. These findings suggest that mutations in the RPT2 domain can disrupt SMARCB1’s ability to support proper SWI/SNF complex assembly and chromatin remodeling, without necessarily leading to complete protein degradation. The close interaction between residues W281 and I315, as shown by structural modeling, and its role in protein function highlight its role in SMARCB1’s tumor suppressor activity through stabilization of the SWI/SNF complex (particularly the cBAF and PBAF subcomplexes).

This is further supported through our analyses of global chromatin regulation and transcription. The W281P and I315R mutants exhibited altered chromatin remodeling patterns, with significant reductions in SWI/SNF activity, as well as changes in gene expression profiles that are consistent with disrupted SMARCB1 function. Notably, the persistence of a small subset of chromatin remodeling activity at AP-1 motifs, despite the mutations, suggests that these variants may not entirely impair the chromatin remodeling activity of SMARCB1. This residual activity may help explain why certain mutations, such as R377H, have been associated with Coffin-Siris Syndrome (CSS) without promoting oncogenesis.

In conclusion, this study provides a comprehensive functional analysis of SMARCB1 variants, identifying key regions and residues involved in its tumor suppressor activity. Our findings suggest that IHC may not be adequate for diagnosing a SMARCB1-deficient cancer, as point mutations can lead to non-functional proteins without complete loss of expression. Furthermore, these findings challenge the assumption that mutation frequency alone predicts pathogenicity and highlight the need for functional assays in assessing the oncogenic potential of specific mutations.

## Methods

### Deep Mutational Scanning

#### DMS Library generation:

SMARCB1 variant library was produced following the principle of the Mutagenesis by Integrated TilEs (MITE) method^40^. The SMARCB1 open reading frame is partitioned into 90-base tiles. DNA Oligos representing all mutations in the space of a tile were synthesized. Each 90 base tile is flanked by 30 base sequences that are complementary to the reference ORF. To avoid interference during tile amplification, oligos for adjacent tiles were synthesized on separate chips. Oligo tile pools were amplified by emulsion PCR using primers to the 30 base flanking regions and purified on a 2% agarose gel. Entry vector cloning is performed on a per tile basis by linearizing the entry vector at the appropriate tile position using Phusion polymerase (New England Biolabs) and primers to the regions flanking the tiles. The linearized vectors were purified on 1% agarose gel and DpnI treated. The DpnI-treated linear plasmid backbones were then mixed with the relevant PCR amplified tile and assembled by *in vitro* recombination with the NEBuilder HiFi DNA Assembly Master Mix (New England BioLabs). The assembly reactions were purified, electroporated into TG1 *E. coli* cells (Lucigen), and recovered for one hour at 37°C in Recovery Media (Lucigen). Aliquots from the transformations were used to inoculate overnight cultures of LB containing 25 μg/mL of Zeocin (ThermoFisher). Cells were harvested by centrifugation and plasmid DNA was isolated using the QIAGEN Midiprep Plus kit (QIAGEN). Plasmid pools, each corresponding to a tile, were verified by sequencing Nextera XT (Illumina). A pool of all pUC57-tile libraries was made with equal weight per variant. This pool was subjected to restriction digest by NheI and BamHI. After purification by 1% agarose gel, the excised linear fragment library is ready for cloning ligation with an expression vector that was pre-processed with NheI and BamHI. The ligated construct was used to transform Stbl4 bacterial cells and plasmid DNA (pDNA) was extracted using QIAGEN Maxi Prep Kits. The resulting pooled pDNA ORF variant library was sequenced via Illumina Nextera XT platform to determine the distribution of variants within the library.

#### Lentiviral Transduction:

293T viral packaging cells were transfected using TransIT-LT1 transfection reagent (Mirus Bio) with three plasmids: the pooled SMARCB1 pDNA library, a packaging plasmid containing gag, pol and rev genes (psPAX2, Addgene), and an envelope plasmid containing VSV-G (pMD2.G, Addgene). Media was changed 6–8 hours after transfection and virus was harvested 24 hours thereafter. In transduction, virus was placed on cells dropwise with polybrene and spun down at 2,000 rpm for 30 minutes at 30°C. Antibiotic selection was performed the following day.

#### ORF extraction from gDNA by PCR:

PCR reactions were set up in 96-well plates using two primers: Forward: 5’-ATTCTCCTTGGAATTTGCCCTT-3’; Reverse: 5’-CATAGCGTAAAAGGAGCAACA-3’. and Q5 DNA polymerase (New England Biolabs). All PCR reactions for each gDNA sample were pooled, concentrated with a PCR cleanup kit (QIAGEN), and separated by gel electrophoresis. Bands of the expected size were excised and DNA was purified using a QIAquick kit (QIAGEN) followed by an AMPure XP kit (Beckman Coulter).

#### Nextera sequencing:

Nextera reactions were performed according to the Illumina Nextera XT protocol. For each sample, we set up 6 Nextera reactions, each with 1 ng of purified ORF DNA. Reactions for each screen sample were indexed with a unique i7/i5 index pair. After the limited-cycle PCR step, the Nextera reaction products were purified with AMPure XP kit. All samples were then pooled and sequenced using an Illumina Nextseq flowcell.

#### Processing next-generation sequencing data:

The NGS reads were processed with a second-generation variant calling software called AnalyzeSaturationMutagenesis (ASMv1.0, as part of GATK, downloadable at https://github.com/broadinstitute/gatk/releases^25^.

### Functional Z-Score Calculation

#### Baseline Calculation Using Silent Mutants:

For each silent mutation, the rolling average and standard deviation of L2FC values were computed within a window of 5 codons (±2 codons flanking the silent mutation). This rolling window approach was implemented to reduce noise from local codon context and sequence-specific effects while ensuring that baseline estimates were still derived from neighboring codons for residues lacking silent mutations. The rolling average represents the expected abundance change for wildtype codons, and the rolling standard deviation reflects the variability of L2FC values for silent codons across the entire DMS library.

The rolling statistics were computed separately for each replicate (G401, BT16, CCLF_PEDS9001T) to capture replicate-specific effects on wildtype abundance. For each codon, the rolling average and standard deviation were calculated as follows:

1. For each codon, we selected all silent codons within the window of ±2 positions.
2. The mean and standard deviation of L2FC values for these silent codons were calculated for each replicate.

#### Z-score Calculation for Variants:

The z-score for each variant was calculated by comparing its L2FC value to the rolling average and standard deviation derived from the silent mutants. For each mutation of interest, the L2FC value from the experimental data was subtracted from the rolling average for the corresponding codon, and the result was divided by the rolling standard deviation. This provides a z-score that reflects how much the variant deviates from the expected abundance changes of silent mutations. Mathematically, the z-score for a given variant was calculated as:

$$Z_{\text{score}}=\frac{L2FC_{\text{variant}}-\text{Rolling}\;{\text{Average}}_{\text{silents}}}{\text{Rolling}\;SD_{\text{silents}}}$$

This calculation was performed separately for each replicate, allowing the assessment of functional impact across different experimental conditions.

#### Z-scores Across Replicates:

For each variant, z-scores were computed for all experimental replicates. The final functional z-score for each variant was calculated by averaging the z-scores across all replicates. The standard deviation of z-scores across replicates was also computed to assess the consistency of variant effects across experiments.

### Nuclear Protein Extraction

Cells were trypsinized and counted using trypan blue exclusion. 25 million cells were spun down at 1,000 rpm for 5 minutes at 4°C. Cells were then washed with cold PBS and spun down at 1,000 rpm for 5 minutes at 4°C. Cell pellets were then resuspended in 5mL of EB0 hypotonic buffer (50mM Tris-HCl, 1mM EDTA, 1mM MgCl_2_, 0.1% NP-40 and supplemented with 1mM PMSF and protease inhibitor), and incubated on ice for 5 minutes. Lysates were then spun down at 4750 rpm for 5 minutes at 4°C. Supernatants were aspirated and remaining pellet was resuspended in 700uL of EB300 (50mM Tris-HCl, 300mM NaCl, 1% NP-40, 1mM EDTA, 1mM MgCl_2_, and supplemented with 1mM PMSF and protease inhibitor). Lysates were incubated on ice and occasionally vortexed for 10 minutes. Lysates were pelleted at 21,000 rpm for 10 minutes and supernatant was collected. Protein concentrations were calculated using a bicinchoninic acid (BCA) assay and frozen at −80°C.

### Lentiviral Transduction for Inducible Cell Lines

Lentiviral particles were generated using 293T cells co-transfected with the gene deliver vector and packaging vectors Δ8.9 and VsVg. More specifically 293T cells were plated in 6 cM plates at a density of 1 million cells per plate in penicillin/streptavidin free DMEM. The following day the 293T cells were transfected. Media was changed with high serum DMEM (30% FBS) and harvested the following two days. The combined viral supernatant was then filtered with 0.45 μm filters. 500 μL of virus was then applied dropwise to target cells in the presence of polybrene (10 μg/mL) and spun down at 2,000 rpm for 30 minutes at 30°C. Antibiotic selection was performed the following day.

### Cell Proliferation Assays

G401 cells were plated in a 6 well plate at a density of 25,000 cells per well and induced with 1μg/mL doxycycline. After 96 hours, cells were trypsinized and counted using trypan blue exclusion. Cells were replated again at a density of 25,000 cells per well and induced again at 1 μg/mL doxycycline. After another 96 hours, cells were trypsinized and counted using trypan blue exclusion. All counts were completed in 2 technical replicates and in biological triplicates for each condition. Cells were then stained using crystal violet at day 10.

### Immunoblots

For experiments using total protein lysate, cells were lysed using 1x RIPA (Cell Signaling Technologies, 9806) with protease inhibitors (coMplete, Roche, 42484600) and phosphatase inhibitors (PhosSTOP, Roche, 04906837001). 4–12% SDS-PAGE gels (SMOBIO Technology, Inc) were transferred onto PVDF (Millipore, IPFL00010) or nitrocellulose membranes (ThermoFisher Scientific, IB23001). Blots were then visualized using Odyssey Classic (LICORbio, Lincoln, NE). Immunoblot was performed using: SMARCB1 (Santa Cruz – sc-166165), β-actin (CST - 8457S), DPF2 (Cell signaling - 71642S), SMARCC2 (Cell signaling - 12760S), SMARCA4 (Santa Cruz- sc-17796). Secondary antibodies: Alexa Fluor^®^ IRDye 680RD goat anti-rabbit (ThermoFisher) or IRDye 800CW goat anti-mouse (LI-COR, Lincoln, NE); with imaging on an Odyssey^®^ scanner (LI-COR). Immunoblot source data are provided in **Supplementary Information**.

### SWI/SNF Co-immunoprecipitation

To perform BAF complex co-immunoprecipitations, BRG1 (G7) antibody was cross-linked with dynabeads (Pierce Protein G Magnetic Beads, Thermo Scientific). Briefly, 5μg of antibody was incubated on a rotator with 50μL of dynabeads for 1 hour at room temperature (RT). Beads were then washed twice with 0.2M NaBorate (pH 9.02) and crosslinked using 6mg/mL Dess–Martin periodinane (DMP) in 0.2M Naborate (pH 9.02) and incubated on a rotator for 45 minutes at RT. Beads were then incubated on a rotator for 1 hour in 0.2M ethanolamine (pH 8.2). Beads were then washed twice with 20mM Tris-HCl, twice with 100mM glycine (pH 2.5), and twice with 100mM Tris-HCl. Beads were then resuspended in IP buffer.

For G401 nuclear extract immunoprecipitations, 800μg of nuclear protein was incubated with crosslinked BRG1-dynabead mixture overnight at 4°C. Beads were then washed 3 times with IP buffer, and 3 times with PBS (1:100 PMSF) and eluted in 50μL of sample buffer (1x NuPAGE LDS Buffer and 100mM DTT). To ensure equivalent loading, 10% of the IP was eluted and analyzed by gel electrophoresis. Silver staining was performed using Pierce^™^ Silver Stain Kit (Lot YC367461). Complex-bound beads were then frozen at −20 °C.

### On-bead/in-solution digestion

For protein digestion, a published protocol was followed^41^. Digestion buffer (50 mM NH4HCO3) was added to the complex-bound beads, and the mixture was then treated with 1 mM dithiothreitol (DTT) at RT for 30 minutes, followed by 5 mM iodoacetimide (IAA) at RT for 30 minutes in the dark. Proteins were digested with 2 μg of lysyl endopeptidase (Wako) at RT for overnight and were further digested overnight with 2 μg trypsin (Promega) at RT. The resulting peptides were desalted with HLB column (Waters) and were dried under vacuum.

### LC-MS/MS

The data acquisition by LC-MS/MS was adapted from a published procedure^42^. Derived peptides were resuspended in the loading buffer (0.1% trifluoroacetic acid, TFA) and were separated on a Water’s Charged Surface Hybrid (CSH) column (150 μm internal diameter (ID) × 15 cm; particle size: 1.7 μm). The samples were run on an EVOSEP liquid chromatography system using the 30 samples per day preset gradient (44 min) and were monitored on a Orbitrap Fusion Lumos Mass Spectrometer (ThermoFisher Scientific, San Jose, CA). The mass was operated in data dependent mode in top speed mode with a cycle time of 3 seconds. Survey scans were collected in the Orbitrap with a 60,000 resolution, 400 to 1600 m/z range, 400,000 automatic gain control (AGC), 118 ms max injection time and rf lens at 30%. Higher energy collision dissociation (HCD) tandem mass spectra were collected in the Orbitrap with a 30,000 resolution, collision energy of 30%, an isolation width of 1.6 m/z, AGC target of 50,000, and a max injection time of 54 ms. Dynamic exclusion was set to 30 seconds with a 10 ppm mass tolerance window.

### MaxQuant

Label-free quantification analysis was adapted from a published procedure^42^. Spectra were searched using the search engine Andromeda, integrated into MaxQuant, against 2022 human UniProtKB/Swiss-Prot database (20,387 target sequences). Methionine oxidation (+15.9949 Da), asparagine and glutamine deamidation (+0.9840 Da), and protein N-terminal acetylation (+42.0106 Da) were variable modifications (up to 5 allowed per peptide); cysteine was assigned as a fixed carbamidomethyl modification (+57.0215 Da). Only fully tryptic peptides were considered with up to 2 missed cleavages in the database search. A precursor mass tolerance of ±20 ppm was applied prior to mass accuracy calibration and ±4.5 ppm after internal MaxQuant calibration. Other search settings included a maximum peptide mass of 6,000 Da, a minimum peptide length of 6 residues, 0.05 Da tolerance for orbitrap and 0.6 Da tolerance for ion trap MS/MS scans. The false discovery rate (FDR) for peptide spectral matches, proteins, and site decoy fraction were all set to 1 percent. Quantification settings were as follows: re-quantify with a second peak finding attempt after protein identification has completed; match MS1 peaks between runs; a 0.7 min retention time match window was used after an alignment function was found with a 20-minute RT search space. Quantitation of proteins was performed using summed peptide intensities given by MaxQuant. The quantitation method only considered razor plus unique peptides for protein level quantitation.

### Mass spectrometry Data Analysis

Statistical analysis was performed with Perseus v2.0.11.0 using LFQ intensity data from single shot mass spectrometry experiments. LFQ data was log2 transformed and proteins with missing values in greater than three samples were removed. Missing values were then imputed based on the assumption of a normal distribution with standard deviation of 0.3 and down shift of 1.8. This down shift was applied to ensure imputed values were biased towards lower intensities. Statistical analysis was then performed using two-sample t-tests comparing each mutant. Proteins with T-test difference > |1.5| and p-value < 0.05 were considered significant.

### RNA sequencing

RNA was extracted from G401 cells following an 8-day induction at 1μg/mL doxycycline for each condition. For mechanistic studies, RNA was collected in biological replicates Libraries were prepared using Illumina TruSeq. Samples were run with at least 20 million paired-end reads using Novoseq 6000 (Illumina). Fastq or BAM files were mapped and aligned using Illumina Dragen v3.7.5 to GrCh38 on Amazon Web Services. Samples were then quantified using salmon through Illumina Dragen v3.7.5. Gene count files were converted to a counts matrix using tximport. The counts matrix was used as input into DESeq2 to evaluate differential gene expression. Normalized read counts matrices were used as input into PCA to visualize clustering between samples. Versions used: R v4.4.2; R Studio 2022.07.2 Build 576; tximport v1.26.1, DESeq2 v1.46.0, ggplot v3.5.1.

### ATAC sequencing

ATAC-seq libraries were made using 50,000 freshly harvested G401 cells with viability > 95%. Tagmentation was performed by incubating cells with Tn5 transposase loaded with Illumina Nextera adaptors for 60 minutes at 37°C. Paired end sequencing for mutant accessibility changes were performed using a Nextseq 2000 (Illumina). Sequencing was completed at the Emory Integrated Genomics Core and samples were sequenced at a depth of at least 50M PE reads. Read trimming, alignment, and duplicate removal was performed using Illumina Dragen v4.2.4. BAM files were then sorted by query name rather than by read coordinate, a step necessary for the subsequent analysis using samtools v1.19.2. The BAM file metadata was then corrected to ensure proper pairing of reads using samtools v1.19.2. Quality control measures were applied to filter out low-quality reads and remove improperly paired or unmapped reads, ensuring only high-quality data were retained for further analysis using samtools v1.19.2. Following this, reads that mapped to non-chromosomal regions and those overlapping known blacklisted regions were excluded using bedtools v2.31.1. These blacklisted regions were sourced from the ENCODE project. After filtering, the BAM file was resorted to restore correct read alignment and indexed to facilitate efficient retrieval and downstream processing using samtools v1.19.2. To enable peak calling, the BAM file was converted to BED format using bedtools v2.31.1. Due to the use of Tn5 transposase during library preparation, read start positions were adjusted according to the known shift introduced by the enzyme, with strand-specific shifts applied. Peak calling was performed using the MACS3 algorithm v3.0.1, which identified regions of significant read enrichment. Finally, the BAM files were converted into BigWig format using deepTools v3.5.4 to allow for efficient visualization and further downstream analysis. Differential accessibility analysis was conducted using DiffBind v3.8.4, with DESeq2 employed for statistical analysis. To compare wildtype to nonsense, we first performed differential accessibility analysis for two separate comparisons: I315I/I315* and WT/W281*. We defined gained peaks as those that showed increased signal in the WT or I315I condition with an FDR <0.05 and Log2FoldChange > 1. Similarly lost peaks were defined as those that showed increased signal in the I315* or W281* condition with an FDR < 0.05 and Log2FoldChange < −1. After independently plotting the significant differentially accessible peaks from each comparison, we visually observed substantial overlap in the regions identified as differentially accessible despite not reaching the significant threshold in DESeq2 analysis. Based on this, we combined the peaks from both sets to create a unified set of significant differentially accessible regions, including both gained and lost regions. ATACseq intensity tornado plots were visualized using computeMatrix and plotHeatmap from deepTools v3.5.4. Replicates were confirmed to be consistent, and subsequently, the bigwigCompare command in deepTools v3.5.4 was used to combine biological duplicates for ATAC-seq signal intensity plots.

Corresponding RNAseq and ATACseq L2FC were assessed by annotating the closest gene associated with each differentially accessible peak using HOMER v4.11.1. The L2FC value associated with each peak was plotted with the corresponding L2FC for the annotated gene. A Fischer’s Exact Test was used to calculate significance using base R.Code for this analysis can be found at https://github.com/ahonglab/SMARCB1_DMS/.

### Molecular Dynamic Modeling

AmberToolsv24 was used for all molecular dynamic simulations. The protein structure was parameterized using the Amber ff19SB force field and solvated in a cubic water box with a 10 Å buffer using the TIP3P water model. Sodium ions were added to neutralize the system, and the final topology and coordinate files were generated for downstream molecular dynamics simulations. Energy minimization of the system was performed using a combination of steepest descent and conjugate gradient methods over 5000 steps. A 10 Å cutoff was applied for non-bonded interactions. The minimized structure was saved for subsequent steps. The system was equilibrated in the NVT ensemble at a target temperature of 300 K using Langevin dynamics for temperature coupling. Subsequent equilibration in the NPT ensemble with a constant pressure of 1 atm was performed. Langevin dynamics were used for temperature coupling with a friction coefficient of 1.0 ps^−1^, and a pressure relaxation time of 2.0 ps was applied. A 10 Å cutoff was applied for non-bonded interactions, and the coordinates were recorded every 100 steps for both equilibration steps.

The production molecular dynamics (MD) simulation was conducted in the NPT ensemble at a target temperature of 300 K and a pressure of 1 atm. A time step of 1 fs was used, and a higher friction coefficient of 5.0 ps^−1^ was applied for temperature coupling. Harmonic positional restraints with a weight of 2.0 kcal/mol·Å^2^ were applied to the alpha carbons (Cα) of residues 1–385 to maintain structural integrity. A 10 Å cutoff was used for non-bonded interactions, and the coordinates were recorded every 100 steps for trajectory analysis. Root mean square fluctuation (RMSF) values were calculated for residues 1–385 to assess residue-level flexibility. The trajectory was analyzed using CPPTRAJ, and RMSF values were computed by residue and output to a data file for further visualization in R. Root mean square deviation (RMSD) of the Cα atoms for residues 1–385 was also computed to evaluate structural stability over time, which was similarly visualized in R. For visualization in VMD, the trajectory was converted from Amber NetCDF format to the CHARMM DCD format. This was achieved by using CPPTRAJ to extract frames from the trajectory and output them in the DCD format for compatibility with VMD. Code for MD simulations can be found at https://github.com/ahonglab/SMARCB1_DMS

### QUANTIFICATION AND STATISTICAL ANALYSIS

GraphPad PRISM 10 and R (v4.4.2) were used to perform statistical analysis. For pairwise comparisons between two groups, a two-tailed student’s unpaired t-test was used. For statistical analysis of ATAC-seq and RNA-seq concordance, observed counts for each quadrant were compared to expected counts under a null hypothesis of uniform distribution using Fisher’s exact test to assess deviations from random association. Statistical analysis of RNA-seq differential expression analysis and ATAC-seq differential accessibility analysis was performed using DESeq2. For RNA-seq analysis, all genes that had an FDR < 0.05 and log2FoldChange > |2| were considered significant. For ATAC-seq analysis, all peaks that had an FDR < 0.05 and log2FoldChange > |1| were considered significant. At least two independent experiments with a least two technical replicates were performed to support statistically analyzed findings.

## Extended Data

## Data Availability

Plasmids herein have been deposited at Addgene (https://www.addgene.org/Andrew_Hong/). The SMARCB1 DMS library is available through the Broad Institute’s Genomic Perturbations Platform (https://www.broadinstitute.org/genetic-perturbation-platform). The RNA-seq, ATAC-seq, and mass spectrometry data supporting the findings of this study will be deposited in the dbGAP database under accession phs003896.v1.p1. Source data are provided with this paper. All other data is available from the corresponding author upon reasonable request.
